# Differential microglial responses to structurally distinct alpha-synuclein polymorphs

**DOI:** 10.1186/s13041-025-01256-0

**Published:** 2025-12-05

**Authors:** Katherine Chang, Jina Kim, Michiyo Iba, Jae-Hyeon Park, Alexandria Beilina, Zulfeqhar A. Syed, Valentina Baena, Liam Horan-Portelance, Mark R. Cookson, Sungyong You, Changyoun Kim

**Affiliations:** 1 Cell Biology and Gene Expression Section, Laboratory of Neurogenetics, National Institute on Aging, National Institutes of Health, Bethesda, MD 20892 USA; 2 Samuel Oschin Comprehensive Cancer Institute, Cedars-Sinai Medical Center, Los Angeles, CA 90048 USA; 3 Departments of Urology and Computational Biomedicine, Cedars-Sinai Medical Center, Los Angeles, CA 90048 USA; 4 Electron Microscopy Core, National Heart, Lung and Blood Institute, National Institutes of Health, Bethesda, MD 20892 USA

## Abstract

Synucleinopathies are age-related neurological disorders which include dementia with Lewy bodies (DLB), Parkinson’s disease (PD), and multiple system atrophy (MSA). A hallmark of these diseases is the pathological accumulation of α-synuclein aggregates, along with sustained neuroinflammatory responses. Recent studies have demonstrated the existence of structurally distinct α-synuclein aggregates in this group of the diseases. While the correlation between specific forms of α-synuclein and distinct pathological characteristics has been extensively studied, their relationship to neuroinflammation remains elusive. Here, we examined the effects of structurally distinct α-synuclein polymorphs on microglial neuroinflammation. Human induced pluripotent stem cells (iPSCs)-derived microglia (iMicroglia, iMG) were treated with α-synuclein polymorphs including EGCG stabilized α-synuclein oligomers (EO), kinetically stable α-synuclein oligomers (KSO), dopamine stabilized α-synuclein oligomers (DO), α-synuclein preformed fibrils (PFF), sonicated α-synuclein preformed fibrils (sPFF), and matured α-synuclein fibrils (Fib). Microglial gene expressions were accessed by transcriptome analysis and Toll-like receptor agonist activities were determined by HEK-Blue TLR reporter assay. Exposures to kinetically stable α-synuclein oligomers and matured α-synuclein fibrils induced the expression of microglial cytokines and chemokines, while other species did not. Microglial transcriptome analysis yielded that all polymorphs commonly induce toll-like receptor (TLR) signaling cascade despite differential transcriptomic phenotypes. Among structurally distinct α-synuclein polymorphs, live cell TLR reporter assay showed that kinetically stable α-synuclein oligomers induce the activities of TLR2 and 4, and sonicated α-synuclein preformed fibril TLR4, relative to the control. These results suggest that structurally distinct α-synuclein polymorphs have likewise distinct neuroinflammatory properties.

**Supplementary Information:**

The online version contains supplementary material available at 10.1186/s13041-025-01256-0.

## Introduction

Synucleinopathies are a categorization of age-related neurological disorders which include dementia with Lewy bodies (DLB), Parkinson’s disease (PD), and multiple system atrophy (MSA) [1, 2]. The progressive accumulation and propagation of α-synuclein in neuronal and non-neuronal cells are key pathological features of the diseases [2, 3]. A large body of evidence supports the existence of cell-autonomous neurotoxicity mediated by α-synuclein aggregates, via disruptions in protein homeostasis, vesicle recycling, and mitochondrial degeneration [2]. Recent studies have also demonstrated non-cell-autonomous neurotoxicity mediated by α-synuclein deposition, including the transmission of α-synuclein between neurons as well as between neurons and various glia, promoting neurotoxic α-synuclein accumulations in neurons and inducing neurotoxic inflammatory responses in microglia [4].

Neuroinflammation is another key pathological feature of synucleinopathies and has been strongly associated with pathogenesis of these diseases [5]. Microglia are resident immune cells in the brain which play a central role in this phenomenon. Neuroinflammatory mechanisms particular to microglia can be caused by a number of factors including misfolded proteins including extracellular α-synuclein aggregates, environmental factors, and genetic factors [2, 5–7]. Recent studies have identified a number of receptors that can interact with extracellular α-synuclein aggregates [8]. Such studies often employ various types of recombinant α-synuclein forms which act as ligands to several other receptors including cluster of differentiation 35 (CD36), macrophage-1 antigen (MAC-1), nicotinamide adenine dinucleotide phosphate (NADPH) oxidase, β1-integrin, and heparan sulfate [9–11]. Among these, we also demonstrated the direct interaction of pattern recognition receptor (PRR) Toll-like receptor 2 (TLR2) with β-sheet-enriched oligomeric forms of neuron-released α-synuclein, including their consequential induction of microglial neuroinflammation and neurotoxicity [12, 13]. Another member of the family, TLR4, has also been shown to interact with recombinant α-synuclein fibrils and play a role in α-synuclein clearance, neuroinflammation, and neurodegeneration in synucleinopathy mouse models [14].

α-Synuclein is known as a typical neuronal cytosolic protein involved in various synaptic homeostatic processes such as synaptic vesicle recycling, and is thought to exist in an unstructured monomeric form [15]. α-Synuclein furthermore is speculated to aggregate into various forms such as oligomers, preformed fibrils, and fibrils under the disease condition [16]. α-synuclein aggregates extracted from synucleinopathy patients have been known to have heterogenous structures interestingly concurrent with the heterogeneity of disease phenotype, even by stage of a so-called singularly defined disease such as PD. While there has been extensive investigation of the potential relationships between structurally distinct α-synuclein polymorphs and distinct pathological and biological properties i.e. seeding differences, toxicity differences, etc., microglial responses against these forms remain largely unknown. Therefore, we comprehensively examined responses to several structurally distinct α-synuclein polymorphs using human induced pluripotent stem cells (iPSCs)-derived microglia (iMicroglia, iMG).

## Materials and methods

### Preparation of α-synuclein aggregates

Recombinant human α-synuclein monomers (#SPR-316, MO), kinetically stable α-synuclein oligomers (#SPR-484, KSO), EGCG stabilized α-synuclein oligomers (#SPR-469, EO), dopamine stabilized α-synuclein oligomers (#SPR-466, DO), and α-synuclein preformed fibrils (#SPR-322, PFF) were purchased from StressMarq Biosciences (Victoria, Canada). To obtain sonicated preformed fibrils (sPFF), PFF were sonicated at room temperature using an ultrasonic homogenizer (Thermo Fisher Scientific, Waltham, MA). To prepare matured α-synuclein fibrils (Fib), recombinant human α-synuclein (3 mg/ml in phosphate-buffered saline, PBS) was incubated at 37 °C for 21 days with shaking at 250 rpm (MaxQ 6000, Thermo Fisher Scientific, Waltham, MA). The protein was centrifuged at 200,000 g for 1 h, and the pellet was resuspended in phosphate buffered saline (PBS).

### Transmission electron microscopy

The ultrastructure of α-synuclein forms was analyzed using negative staining and transmission electron microscopy (TEM). Carbon-coated copper grids (300 mesh, Electron Microscopy Sciences, # CF300H-Cu-25) were glow-discharged, and an aliquot of 4 μl of the sample was applied to the grid and allowed to adsorb for 10 min at room temperature followed by gentle blotting with a filter paper (Sigma-Aldrich, # WHA1001090). Grids were washed twice on droplets of phosphate-buffered saline (PBS, pH 7.2) followed by deionized water 3 times for 30 s each and stained with 2% (w/v) uranyl acetate (Electron Microscopy Sciences, #22400–2) for 30 s. Excess stain was blotted off with filter paper, and grids were air-dried at room temperature for 5 min. Imaging was performed using JEOL JEM 1200 EXII TEM operating at 80 kV and equipped with AMT XR-60 digital camera. The diameter of α-synuclein oligomers and width and length of fibrillar species were determined by ImageJ Fiji.

### Immunoblot analysis

Two nanograms of α-synuclein monomer, oligomers, or fibrillar species were separated with 4–12% Bis–Tris SDS-PAGE gels by electrophoresis and transferred onto PVDF membranes using a semidry Trans-Blot Turbo Transfer System (Bio-Rad, Hercules, CA). The membranes were blocked with Odyssey blocking buffer (LI-COR Biosciences, Lincoln, NE) and subsequently incubated with primary antibodies against total or conformation-specific α-synuclein and followed by fluorescent secondary antibodies (Supplementary Table 1). Fluorescence signal detection and densitometric analysis were performed using ODYSSEY CLx (LI-COR Biosciences) and Image Studio software (LI-COR Biosciences).

### Thioflavin T assay

The procedure for Thioflavin T assay was described elsewhere [17]. Briefly, 10 μM of protein samples were incubated with 10 μM of Thioflavin T and incubated for 5 min at room temperature. Thioflavin T fluorescence was measured by SpectraMax GEMINI EM (Molecular devices, Sunnyvale, CA) at an excitation of 450 nm and emission at 490 nm.

### Differentiation of human iPSCs into human microglia (iMicroglia, iMG)

A18945 human iPSC lines were maintained in Essential 8™ Medium (Thermo Fisher Scientific) with supplement (Thermo Fisher Scientific), and 10 μM of ROCK inhibitor (StemCell Technologies, Vancouver, Canada) was added for 1 day after passaging. Then iPSCs lines were replenished with E8 media every day until reaching confluency and ready for passaging.

For the microglia differentiation, cells were cultured without ROCK inhibitor for at least 2 days prior to the initiation of differentiation. On day 1, 10,000 cells per well were plated in V-bottom, ultra-low attachment 96-well plates (S-bio, Constantine, MI) in Embryoid Body Media (EBM) containing E8 Medium, 10 μM ROCK inhibitor, 50 ng/mL BMP-4 (Thermo Fisher Scientific), 20 ng/mL SCF (Thermo Fisher Scientific), and 50 ng/mL VEGF-121 (Thermo Fisher Scientific). On day 2, remove 80 ul of the EBM media and replace with 100 ul of fresh media without ROCK inhibitor into each 96 well. On day 4, remove most of the EBM media and transfer 192 EBs from 96 well plates using VWR® Wide Bore Pipette Tips (Avantor Inc, Radnor, PA) to collect the EB’s in a reservoir, and then transfer the EBs in a 50 ml Falcon tube. Media were carefully removed and replaced with 25 ml of Microglia Progenitor Media (MPM) containing X-VIVO 15 Medium (Lonza, Basel, Switzerland), 2 mM GlutaMax, 55 mM beta-Mercaptoethanol, 100 ng/mL M-CSF (Thermo Fisher Scientific), and 25 ng/mL IL-3 (Thermo Fisher Scientific). The EBs adhered in a fibronectin (Sigma Aldrich)-coated T-75 flasks within 1–2 days. Fibronectin was diluted to 10 µg/mL in PBS and incubated in the flasks for 24 h at 37 °C. After incubation, the flasks were washed three times with water before adding the cells, and media was changed every other day with fresh MPM media [18].

For maturation of microglia iMG, precursor cells were collected by centrifugation at 300 × g for 5 min and then seeded in Microglia Maturation Media (MMM). Specifically, 0.6–0.7 million cells were plated per well in a non-coated 6-well plate. MMM containing Advanced RPMI 1640 Medium (Thermo Fisher Scientific), 2 mM GlutaMax (Thermo Fisher Scientific), 100 ng/mL IL-34 (Thermo Fisher Scientific), and 10 ng/mL GM-CSF (Thermo Fisher Scientific). The cells were further differentiated for an additional 10–12 days, with MMM media changes every other day.

All detailed information for reagents is displayed in Supplementary Table 2.

### Generation of monocytes

Human Bone Marrow Mononuclear Cells (BM-MNCs) were obtained from StemCell Technologies (#70001). To generate monocytes, BM-MNCs were cultured for 4 days in RPMI 1640 medium supplemented with 10% fetal bovine serum (FBS) containing 20 ng/mL macrophage colony-stimulating factor (M-CSF). Cultures were maintained in non-adherent Petri dishes to support monocyte differentiation.

### Cell culture and treatments

iMG or human monocytes was treated with indicated concentrations of control, α-synuclein MO, α-synuclein EO, α-synuclein KSO, α-synuclein DO, α-synuclein PFF, α-synuclein sPFF, or α-synuclein fibril for indicated hours. To modulate TLR2 activity, iMG were pre-treated with control IgG (1 μg/mL, InvivoGen, San Diego, CA) or anti-TLR2 (T2.5, 1 μg/mL, InvivoGen) for 30 min, and the cells were exposed to control, α-synuclein KSO, or α-synuclein Fib for 4 h.

Primary postnatal microglia were isolated from wild type mice pups (P_0-1_). The procedure for primary microglia culture has been described elsewhere [13]. Isolated microglia were seeded onto poly-D-lysine (PDL)-coated 12 well culture plates and the cells were treated with 200 nM of control, α-synuclein MO, α-synuclein EO, α-synuclein KSO, α-synuclein DO, or α-synuclein PFF for four hours.

HEK-Blue hTLR2 Cells (#hkb-htlr2), HEK-Blue hTLR3 Cells (#hkb-htlr3), HEK-Blue hTLR4 Cells (#hkb-htlr4), HEK-Blue hTLR5 Cells (#hkb-htlr5), HEK-Blue hTLR7 Cells (#hkb-htlr7), HEK-Blue hTLR8 Cells (#hkb-htlr8), and HEK-Blue hTLR9 Cells (#hkb-htlr9) were purchased from InvovoGen. Cells were maintained according to the manufacturer’s instructions.

### Quantitative polymerase chain reaction (qPCR)

Total RNA was extracted from cells utilizing an RNeasy mini kit (Qiagen, Hilden, Germany). A total of 0.1–0.5 μg of RNA was reverse transcribed using SuperScript VILO cDNA synthesis kit (Thermo Fisher Scientific) according to the manufacturer’s instructions.

Quantitative real-time PCR was performed using TaqMan Fast Advanced Master Mix (Thermo Fisher Scientific) with various gene-specific primers (Supplementary Table 3). DNA amplification was measured by the StepOnePlus real-time PCR system (Thermo Fisher Scientific). Relative gene expression was calculated according to the ΔΔCt method relative to β-actin.

### Enzyme-linked immunosorbent assay (ELISA)

To determine microglial secretion of cytokines and chemokines, iMG were treated with either control or α-synuclein polymorphs for 24 h. The culture media was harvested and centrifuged at 10,000 g for 5 min to remove cell debris. The levels of cytokines and chemokines in the culture media (50 μL per each sample) were measured using ELISA kits, including the Human TNF alpha ELISA kit, Human IL-6 ELISA kit (Thermo Fisher Scientific), Human CCL2/MCP-1 ELISA kit, Human CCL4/MIP-1 beta ELISA kit (R&D Systems, Minneapolis, MN). The levels of human TNFα, human IL-6, human CCL2, and human CCL4 were determined by SpectraMax M5e microplate reader (Molecular Devices).

### iMicroglial transcriptome analysis

Total RNA was isolated from iMG exposed to control, α-syn EO, KSO, DO, PFF, sPFF, or Fib for four hours (*n* = 4 per group), and RNA sequencing was performed by Psomagen (Rockville, MD). RNA sequencing raw data was first assessed for quality using FastQC (v0.11.9) [19]. Adapter sequences were subsequently removed from the paired-end reads using Trim Galore (v0.6.4_dev) [20]. The cleaned reads were then aligned to the human reference genome (GRCh38) using STAR aligner (v2.7.5c) [21], and transcript abundance was quantified with RSEM (v1.3.3) [22].

Raw count data were preprocessed following a series of filtering and normalization steps. First, features with a read count of more than 10 in all 32 samples were kept, resulting in a total of 12,309 gene features. Subsequently, normalization was performed using the Trimmed Mean of M-values (TMM) method [23]. Finally, log2 transformation was applied to the normalized expression values. The analysis of differentially expressed genes (DEGs) was performed using the integrated hypothesis testing method (t-test and median difference test for each gene) [24]. Stouffer’s method was used to combine the two p-values into an overall p-value, which was adjusted for multiple testing using Benjamini–Hochberg method to control the false discovery rate (FDR). DEGs were selected with FDR ≤ 0.05 and the absolute value of Log2 fold-change (log_2_FC) ≥ 0.58. For heatmap visualizations, we used the pheatmap package (v1.0.12) [25] to illustrate gene expression patterns across samples.

### Functional enrichment analysis

The Gene Ontology (GO) and Kyoto Encyclopedia of Genes and Genomes (KEGG) pathway enrichment analyses were performed using gprofiler2 (v0.2.3) [26], and statistical significance was determined based on adjusted Fisher’s exact test p-value (FDR < 0.05). Enrichment results were further examined with a specific focus on immune-related pathways. For comparative analysis of multiple enriched pathways, radar charts were created using the fmsb package (v0.7.6) [27]. The adjusted p-values were presented within predefined minimum and maximum ranges of radar plots.

### HEK-Blue TLR reporter assay

To determine TLR agonist effect of α-synuclein aggregates, HEK-Blue hTLR reporter cells and HEK-Blue TLR reporter assay were utilized (InvivoGen). The cells were treated with indicated concentrations of negative control, agonists, α-synuclein monomer, or α-synuclein aggregates. TLR agonists were obtained from InvivoGen and used as positive controls (Supplementary Table 4). HEK-Blue TLR reporter assay was performed according to manufacturer’s instructions.

### Statistical analysis

All data are presented as the mean ± SEM. Statistical analysis was performed using GraphPad Prism 10 (GraphPad, San Diego, CA). Statistical analyses were conducted using unpaired *t* test and one-way ANOVA followed by Tukey’s multiple comparison tests. The number of independent experiments in in vitro assays and number of sample counts are represented by N. No sample was excluded from the analysis.

## Results

### Characterization of structurally distinct α-synuclein aggregates

**Fig. 1:**
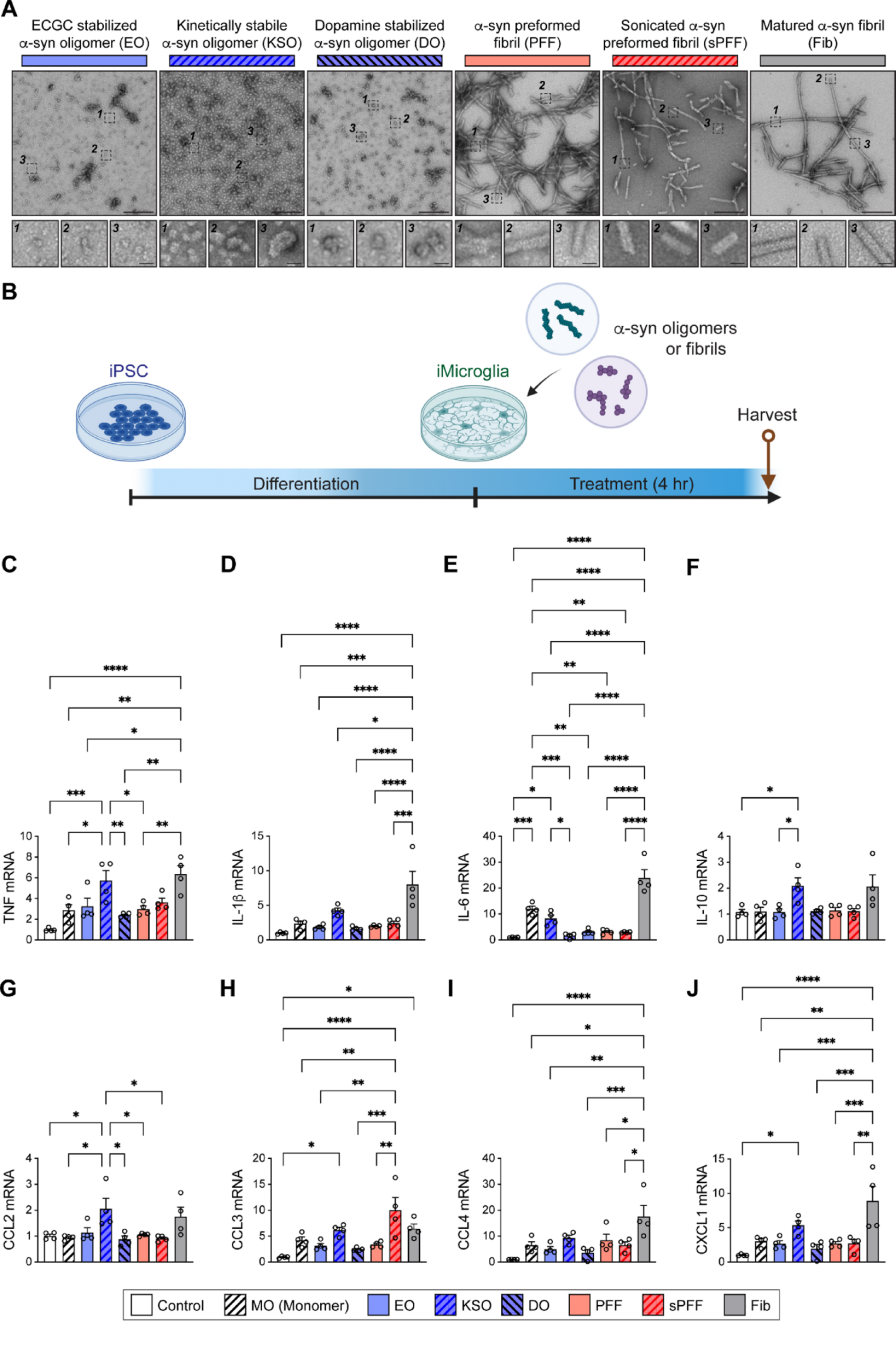
Microglial inflammatory gene expression induced by α-synuclein polymorphs. **A** Electron microscopy analysis of α-synuclein EO, KSO, DO, PFF, sPFF, and Fib. Scale bars 200 nm (Upper panel) and 20 nm (Lower panel). **B** Experimental scheme. iPSC-differentiated iMG were exposed to control, α-synuclein MO, EO, KSO, DO, PFF, sPFF, or Fib for 4 h. **C–J** iMG expressions of TNF (**C**), IL-1β (**D**), IL-6 (**E**), IL-10 (**F**), CCL2 (**G**), CCL3 (**H**), CCL4 (**I**), and CXCL1 (**J**) were determined by quantitative polymerase chain reaction (PCR). Error bars represent SEM. One-way ANOVA and Tukey’s multiple comparison test (*n* = 4 per group). *p < 0.05, **p < 0.01, ***p < 0.001, ****p < 0.0001

We first characterized the structures of recombinant α-synuclein polymorphs via transmission electron microscopy (TEM), immunoblot analysis with conformationally preferential antibodies, and thioflavin T assay (Fig. 1A and Supplementary Fig. 1). The overall morphologies of EGCG stabilized α-synuclein oligomers (EO), kinetically stable α-synuclein oligomers (KSO), dopamine stabilized α-synuclein oligomers (DO), α-synuclein preformed fibrils (PFF), sonicated α-synuclein preformed fibrils (sPFF), and matured α-synuclein fibrils (Fib) were analyzed via negative-staining TEM (Fig. 1A and Supplementary Fig. 1A, B, C). Consistent with previous observations [28, 29], we observed oligomeric species to have spherical-like morphologies and the fibrillar species more filament-like structures (Fig. 1A). The distribution analysis of α-synuclein oligomer diameters demonstrated that KSO samples tended to have oligomers larger in diameter than either EO or DO (Supplementary Fig. 1A). The distribution analysis of α-synuclein fibrillar length demonstrated that both PFF and Fib tend to have larger spread and sPFF tended shorter (Supplementary Fig. 1B). The width of PFF tended to remain unaffected by sonication while Fib had thicker and more homogenous widths (Supplementary Fig. 1C).

We next performed a biochemical characterization of α-synuclein aggregates utilizing immunoblot analysis (Supplementary Fig. 1D, E, F, G). α-synuclein aggregates including MO, EO, KSO, DO, PFF, sPFF, and Fib were separated by immunoblot analysis and the blots were incubated with various types of α-synuclein antibodies against total and conformationally preferential α-synuclein. The analysis revealed the detection of different populations of α-synuclein polymorphs among the aggregates. Incubation with anti-total α-synuclein (SYN1) indicates monomeric and high molecular weight aggregated α-synuclein in the samples. Interestingly, KSO and DO tended to concentrate at higher molecular weights, while EO displayed a broader range of molecular weights, and PFF and sPFF were found at a lower range, with clear expected bands around 14 kDa as expected for the monomeric sample. Although thioflavin T fluorescence was significantly lower in sPFF compared to PFF, their separation patterns remained similar. Incubation with conformationally preferential anti-α-synuclein (2F11) identified immunoreactivity against MO and all other forms besides EO and DO (Supplementary Fig. 1E). Incubation with another conformationally preferential anti-α-synuclein (5G4) also showed similar immunoreactivities against MO and forms, compared to anti-total α-synuclein immunoblot (Supplementary Fig. 1F). However, immunoreactivity against PFF was absent for sPFF, and similar results were seen with monoclonal antibody LB509, while other forms were immunoreactive with antibody 5G4 (Supplementary Fig. 1F, G).

To gain insight into the structural features of the α-synuclein aggregates, we assessed their secondary structure content utilizing a thioflavin T (ThioT) assay (Supplementary Fig. 1H). As expected, the levels of ThioT fluorescence were significantly higher in both PFF and Fib relative to the α-synuclein monomer (MO) and oligomeric species. Sonication of PFF (sPFF) significantly reduced ThioT reactivity of PFF, suggesting a decrease in β-sheet structure of PFF with sonication.

Taken together, these results demonstrate that the different α-synuclein aggregates employed in the current study have distinct structural characteristics.

### Distinct microglial responses against α-synuclein polymorphs

We next investigated microglial inflammatory responses to structurally distinct α-synuclein polymorphs. To this end, we employed human induced pluripotent stem cell (iPSC)-derived microglia (iMicroglia, iMG). To assess the microglial response to α-synuclein polymorphs, iMG were treated with various concentrations of α-synuclein KSO, sPFF, and Fib for different time points (Supplementary Fig. 2). The expression levels of proinflammatory cytokines *TNF* and *IL-6* were elevated following treatment with 200 nM and 1000 nM of α-synuclein KSO or Fib (Supplementary Fig. 2A, B, E, F). In contrast, *TNF* and *IL-6* expression was increased only at 200 nM of α-synuclein sPFF (Supplementary Fig. 2C, D). *TNF* expression began to increase at 4 h after treatment with α-synuclein KSO, sPFF, and Fib (Supplementary Fig. 2G, I, K). *IL-6* expression also started to increase at 4 h after treatment with α-synuclein sPFF and Fib (Supplementary Fig. 2J, L), whereas a significant increase in *IL-6* was observed at 24 h following α-synuclein KSO treatment (Supplementary Fig. 2H). Interestingly, the increase in *IL-6* expression induced by α-synuclein Fib was highest at the 4-h time point and declined at later time points (Supplementary Fig. 2L).

To compare microglial inflammatory gene expression in response to different α-synuclein polymorphs, cells were treated with control, α-synuclein MO, EO, KSO, DO, PFF, sPFF, or Fib for 4 h (Fig. 1B). The expressions of proinflammatory cytokines and chemokines were determined by quantitative mRNA expression analysis (Fig. 1C, D, E, H, I, J). The expression of *TNF* and *CXCL1* were significantly increased by KSO and Fib treatments, compared to control treatment (Fig. 1C, J). The expression of *IL-1β* and *CCL4* were only significantly increased in iMG exposed to Fib (Fig. 1D, I). Interestingly, the expression of *IL-6* was increased by MO and Fib treatments (Fig. 1E). The expression of *IL-10* and *CCL2* were only significantly increased in iMG treated with KSO (Fig. 1F, G). The expression of *CCL3* was increased in iMG treated with KSO, sPFF, and Fib (Fig. 1H). Microglial secretions of TNF, CCL2, and CCL4 were increased by Fib treatment, whereas IL-6 secretion was elevated following both KSO and Fib treatments (Supplementary Fig. 3A, B, C, D).

To validate these findings, we treated primary mouse microglia with control, α-synuclein MO, EO, KSO, DO, or PFF (Supplementary Fig. 4). The expression of *Tnf* and *Il-1β* were only increased by KSO, while *Il-6* mRNA expression was increased by treatments of α-synuclein oligomers, including EO, KSO, and DO (Supplementary Fig. 4A, B, C). However, the expression of chemokines, such as *Ccl2*, *Ccl3*, *Ccl4*, and *Ccl5* were not induced by α-synuclein treatments in primary mouse microglia (Supplementary Fig. 4D, E, F, G). To investigate whether α-synuclein polymorphs induce inflammatory responses in immune cells other than microglia, we treated human monocytes with various α-synuclein polymorphs (Supplementary Fig. 5). Human bone marrow mononuclear cells were differentiated into monocytes and treated with α-synuclein polymorphs for 4 h (Supplementary Fig. 5A). The expression of proinflammatory cytokines and chemokines including TNF, IL-1β, IL-6, CCL3, CCL4, CCL5, and CXCL1 was increased exclusively in response to α-synuclein Fib treatment in human monocytes (Supplementary Fig. 5B, C, D, F, H, I, J, K). CXCL10 expression was induced by both α-synuclein KSO and Fib treatments (Supplementary Fig. 5K), whereas IL-10 and CCL2 expression levels were not altered by any α-synuclein polymorph treatment (Supplementary Fig. 5E, G).

**Fig. 2:**
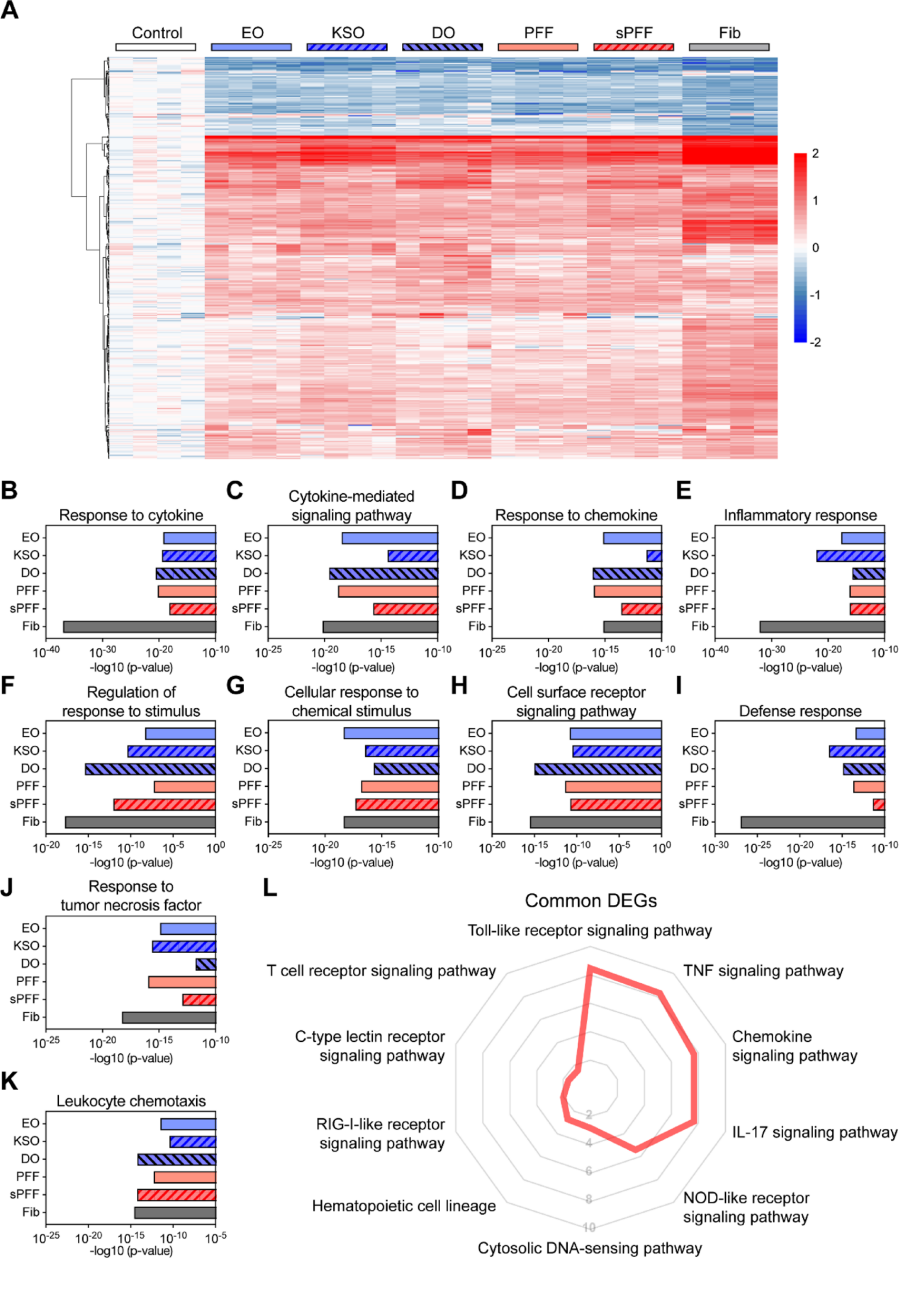
Transcriptomic analysis of microglia exposed to α-synuclein polymorphs. iPSC-differentiated iMicroglia were exposed to control, α-synuclein MO, EO, KSO, DO, PFF, sPFF, or Fib for 4 h (*n* = 4 per group). Total RNA extracted from iMG was analyzed by RNA sequencing. **A** Heatmap of the differentially expressed genes in the group of comparisons. Red and blue represent down- and up-regulation (Cut-off criteria: FDR < 0.05 and the absolute value of log_2_FC ≥ 0.58). **B–K** GO:BP enrichment analysis (Fisher’s exact test *p*-value with FDR correction < 0.05). Enriched GO:BP pathways in iMG expose to α-synuclein aggregates, response to cytokine (**B**), cytokine-mediated signaling pathway (**C**), response to chemokine (**D**), inflammatory response (**E**), regulation of response to stimulus (**F**), cellular response to chemical stimulus (**g**), cell surface receptor signaling pathway (**H**), defense response (**I**), response to tumor necrosis factor (**J**), and leukocyte chemotaxis (**K**). The *y*-axis displays treatments, and the *x*-axis shows the -log_10_ *p*-value. **L** Radar plot for p-values of enriched KEGG immune pathway of common up-regulated genes among the group of comparisons

To gain a comprehensive understanding of microglial responses against different α-synuclein polymorphs exposure, we next performed transcriptome analysis. Microglial differentially expressed genes (DEGs) were determined by comparison of α-synuclein treated cells and vehicle treated control cells. We found 109 DEGs (91 up-regulated and 18 down-regulated) in EO treated cells, 131 DEGs (115 up-regulated and 16 down-regulated) in KSO treated cells, 144 DEGs (131 up-regulated and 13 down-regulated) in DO treated cells, 107 DEGs (86 up-regulated and 21 down-regulated) in PFF treated cells, 137 DEGs (116 up-regulated and 21 down-regulated) in sPFF treated cells, and 297 DEGs (240 up-regulated and 57 down-regulated) in Fib treated cells, compared to control cells (Fig. 2A).

We next performed functional enrichment analysis to identify enriched gene ontology biological processes (GO:BP) (Fig. 2B, C, D, E, F, G, H, I, J, K). Interestingly, we found that the up-regulated genes from all comparison were enriched in microglial inflammatory response ontologies, such as response to cytokine, cytokine-mediated signaling pathway, response to chemokine, inflammatory response, regulation of response to stimulus, cellular response to chemical stimulus, cell surface receptor signaling pathway, defense response, response to tumor necrosis factor, and leukocyte chemotaxis. Among the comparisons, α-synuclein fibril treated microglia showed greater response to cytokine, inflammatory response, and defense response, compared to other comparisons (Fig. 2B, E, I). Functional enrichment analysis to identify cellular pathway (KEGG) identified ten enriched immune-related pathways, such as toll-like receptor (TLR) signaling pathway, TNF signaling pathway, chemokine signaling pathway, IL-17 signaling pathway, NOD-like receptor (NLR) signaling pathway, cytosolic DNA-sensing pathway, hematopoietic cell lineage, RIG-I-like receptor (RLR) signaling pathway, C-type lectin receptor (CLR) signaling pathway, and T cell receptor (TCR) signaling pathway in the common up-regulated genes in each comparison (Fig. 2L). In contrast, down-regulated genes were not enriched in either cell processes or cellular pathways.

To gain more distinguished information from functional enrichment analysis, we displayed the enriched immune pathways of DEGs utilizing radar plot (Supplementary Fig. 6). In microglia exposed to α-synuclein EO, DO, PFF, and sPFF, DEGs were strongly enriched in chemokine signaling pathway, IL-17 signaling pathway, TNF signaling pathway, TLR signaling pathway, NLR signaling pathway, compared to control cells (Supplementary Fig. 6A, C, D, E). However, the DEGs of theses were not significantly enriched in cytosolic DNA-sensing pathway, RLR signaling pathway, hematopoietic cell lineage, CLR signaling pathway, and TCR signaling pathway (Supplementary Fig. 6A, C, D, E). In contrast, DEGs from KSO and Fib were strongly enriched in TNF signaling pathway, but also in chemokine signaling pathway, IL-17 signaling pathway, TLR signaling pathway, and NLR pathway (Supplementary Fig. 6B, F). Interestingly, DEGs from KSO are enriched in CLR signaling pathway, RLR signaling pathway, and cytosolic DNA-sensing pathway, while DEGs from α-synuclein fibril are enriched in hematopoietic cell lineage and CLR signaling pathway (Supplementary Fig. 6B, F).

Taken together, although functional enrichment analysis of DEGs from microglia exposed to structurally distinct α-synuclein polymorphs demonstrated some similar cellular process and pathways, there were also distinct pathways triggered by specific polymorphs, suggesting access to specific signaling pathways.

### Investigation of agonist role of α-synuclein polymorphs in TLR activity

TLRs are strongly associated with pathogenesis of synucleinopathies and have been known to interact with certain types of extracellular α-synuclein polymorphs [30, 31]. Among glial cells, TLRs are mostly expressed in microglia (Supplementary Fig. 7). Specifically, TLR2, 3, 5, and 7 are highly expressed in microglia while TLR4, 8, and 9 showed lower expression in these cells (Supplementary Fig. 7). In the current study, TLR signaling pathway was determined most strongly enriched in the DEGs from microglia exposed to α-synuclein polymorphs (Fig. 2L). Thus, we next investigated the capability of structurally distinct α-synuclein polymorphs to induce TLR activity utilizing human TLR reporter cells (HEK-Blue-hTLR), ectopically expressing human TLR and monitoring reporter system.

**Fig. 3:**
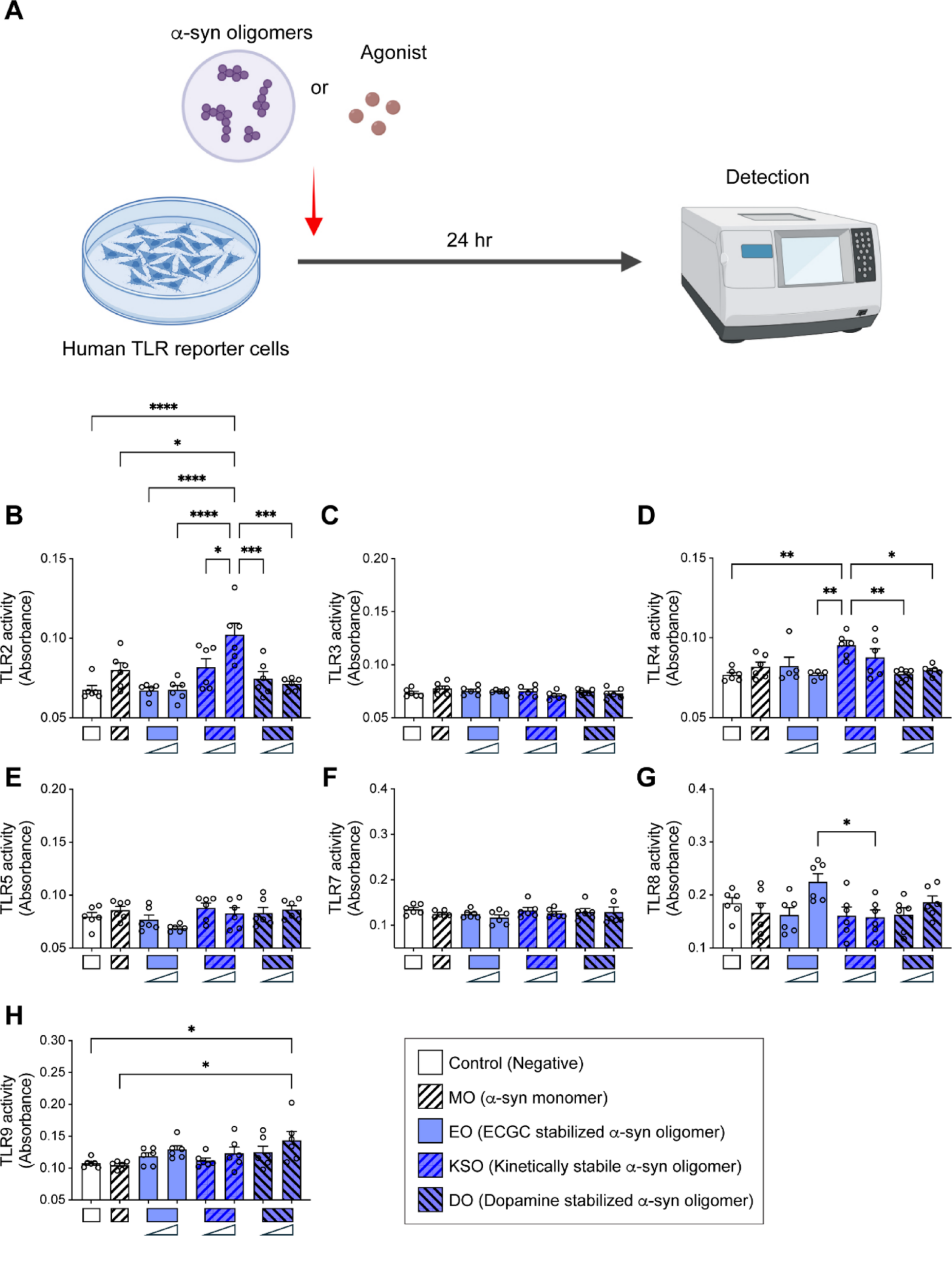
TLR agonist ability of α-synuclein oligomers. **A** Schematic illustration of experiment. HEK-Blue hTLR reporter cells were treated with low (100 nM) and high (1 μM) concentrations of α-synuclein oligomers and α-synuclein MO. **B**–**H** After 24 h of incubation, the activities of TLR2 (**B**), TLR3 (**C**), TLR4 (**D**), TLR5 (**E**), TLR7 (**F**), TLR8 (**G**), and TLR9 (**H**) were determined by TLR activity reporter assay (*n* = 6 per group). Vehicle and TLR agonist were used as negative and positive controls, respectively. Error bars represent SEM. One-way ANOVA and Tukey’s multiple comparison test. *p < 0.05, **p < 0.01, ***p < 0.001, ****p < 0.0001

**Fig. 4:**
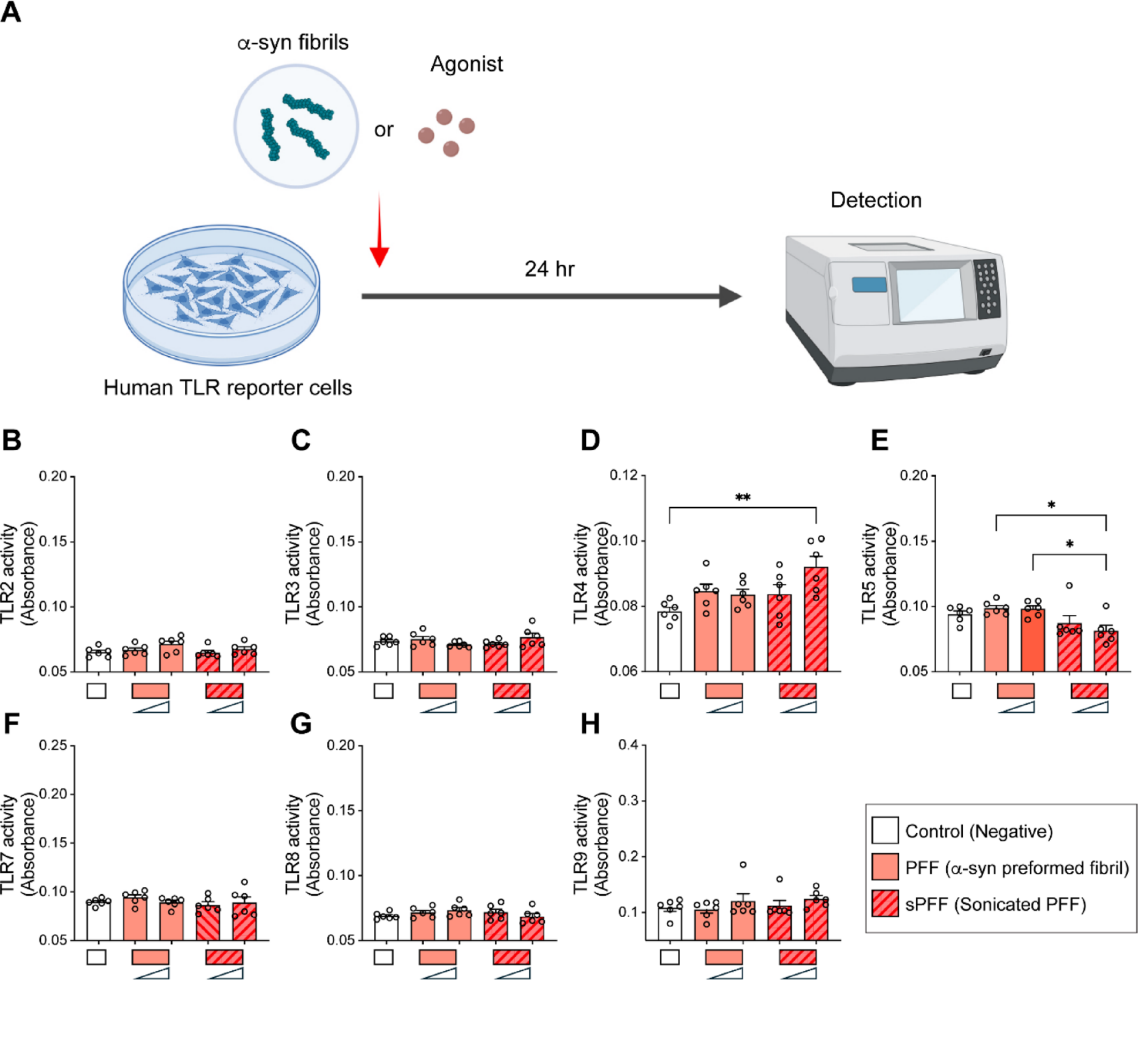
TLR induction ability of α-synuclein fibrillar species. **A** Experimental scheme. HEK-Blue hTLR reporter cells were treated with low (100 nM) and high (1 μM) concentrations of α-synuclein PFF and α-synuclein sPFF. **B–H** The activities of TLR2 (**B**), TLR3 (**C**), TLR4 (**D**), TLR5 (**E**), TLR7 (**F**), TLR8 (**G**), and TLR9 (**H**) were determined by TLR activity reporter assay after a 24-h incubation (*n* = 6 per group). Vehicle and TLR agonist were used as negative and positive controls, respectively. Error bars represent SEM. One-way ANOVA and Tukey’s multiple comparison test. *p < 0.05, **p < 0.01

HEK-Blue-hTLR2, 3, 4, 5, 7, 8, and 9 cells were treated with indicated concentrations of either α-synuclein MO or oligomers, and TLR activities were determined post 24 h treatment (Fig. 3A). TLRs specific agonists and vehicle are used as positive and negative controls, respectively (Supplementary Fig. 8). Interestingly, treatment of α-synuclein KSO significantly increased TLR2 activity in a dose dependent manner (Fig. 3B). TLR4 activity was also increased with low doses of KSO treatment but did not increase with high dose treatment (Fig. 3D). However, the activities of TLR3, 5, 7, 8, 9 were not altered by KSO treatment (Fig. 3C, E, F, G, H). The activities of TLRs were not affected by MO, EO, and DO, except TLR9 (Fig. 3B, C, D, E, F, G, H). The activity of TLR9 was only significantly increased by high dose treatment of DO (Fig. 3H). HEK-Blue-hTLR2, 3, 4, 5, 7, 8, and 9 cells were treated with indicated concentrations of either PFF, or sPFF for 24 h (Fig. 4A). TLR activity was not generally induced by PFF or sPFF treatment, except TLR4 (Fig. 4B, C, D, E, F, G, H). Interestingly, the activity of TLR4 was significantly increased by high dose treatment of sPFF, while it was not induced by the same concentration of PFF (Fig. 4D).

**Fig. 5:**
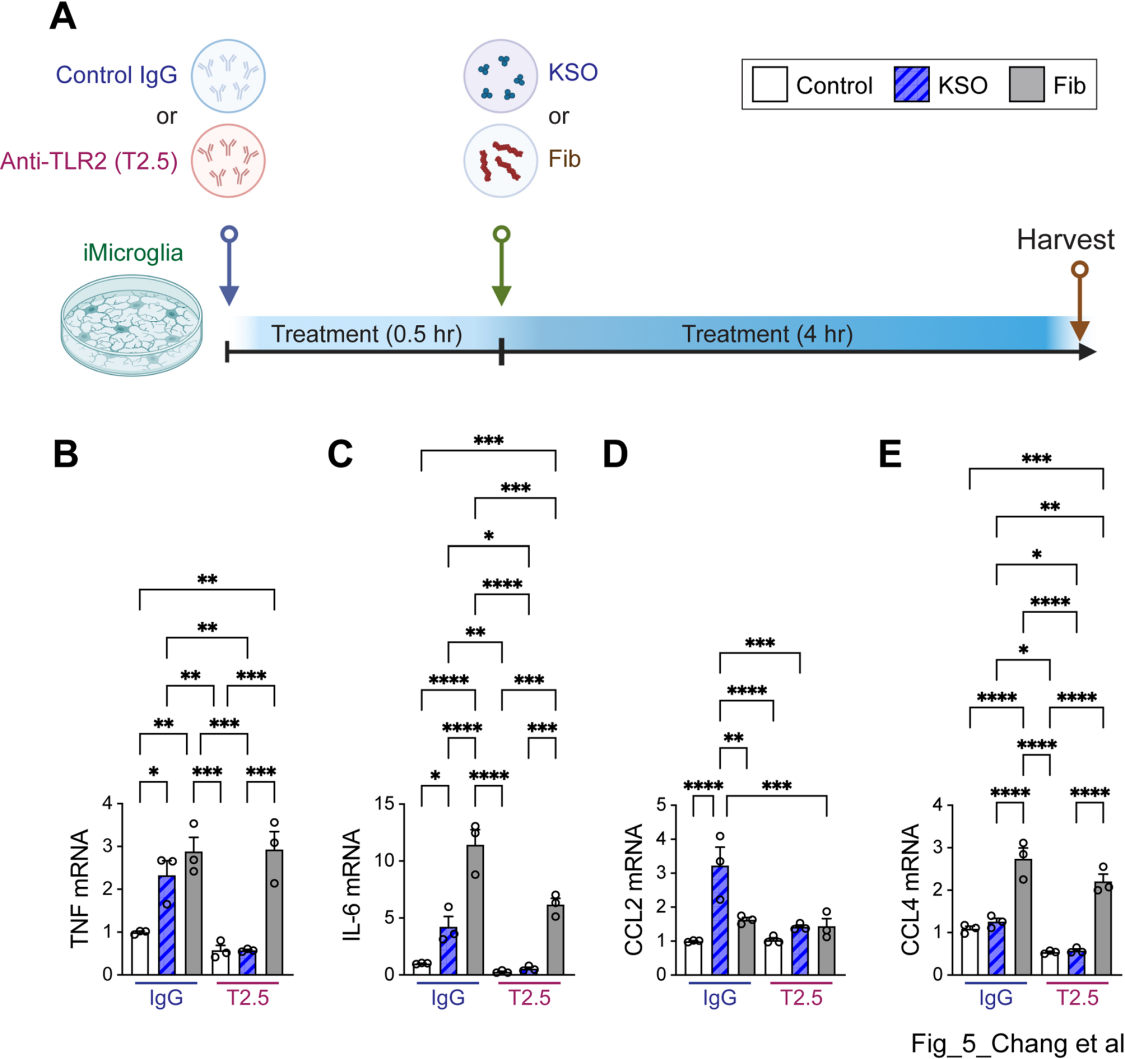
Inhibition of TLR2 reduces α-synuclein polymorphs-induced inflammatory gene expression in iMicroglia. **A** Experimental scheme. iMG were pretreated with either control IgG (1 μg/mL) or anti-TLR2 antibody (T2.5, 1 μg/ML) for 30 min, followed by treatment with control, α-synuclein KSO, or Fib for 4 h. **B–E** Expression levels of TNF (**B**), IL-6 (**C**), CCL2 (**D**), and CCL4 (**E**) in iMG were assessed by quantitative PCR. Error bars represent SEM. One-way ANOVA and Tukey’s multiple comparison test (*n* = 3 per group). *p < 0.05, **p < 0.01, ***p < 0.001, ****p < 0.0001

According to TLR reporter cell analysis, sPFF was capable of activating TLR4 (Fig. 4D). However, due to the lack of a significant microglial response following low-dose (200 nM) and short-duration (4 h) sPFF treatment, we stimulated iMG with α-synuclein KSO in the presence of either control IgG or anti-TLR2 (T.2) antibody to validate the interaction between α-synuclein KSO and TLR2 in microglia (Fig. 5A). iMG were pre-treated with IgG or T2.5 for 30 min, then exposed to α-synuclein KSO or Fib for 4 h. α-synuclein Fib was used as a positive control. The expression levels of TNF, IL-6, and CCL2 were significantly increased following α-synuclein KSO and Fib treatments in the presence of IgG (Fig. 5B, C, D). However, pre-treatment with T2.5 significantly reduced the expression of these genes in α-synuclein KSO-treated iMG, while gene induction by α-synuclein Fib was unaffected by T2.5 pre-treatment (Fig. 5B, C, D). Consistent with Fig. 1I, CCL4 expression was not elevated by α-synuclein KSO treatment in iMG, whereas it was significantly increased by α-synuclein Fib (Fig. 5E).

Taken together, these observations support that structurally distinct α-synuclein polymorphs have different TLR agonist capabilities.

## Discussion

Abnormal deposition of α-synuclein is a commonality of synucleinopathies [32]. Accumulating evidence indicates that α-synuclein exists in varying conformations in the brains of patients [33, 34]. For example, a high-resolution structural study of isolated α-synuclein aggregates from PD/DLB and MSA patients post-mortem demonstrated conformationally structurally distinct forms [35]. In addition, α-synuclein isolated from oligodendrocytes were structurally distinct from α-synuclein isolated from neurons under disease conditions [36]. Furthermore, it has been shown that inoculation of animal models with distinct α-synuclein aggregates isolated from synucleinopathy patients with heterogeneous disease phenotypes induced likewise distinct α-synuclein neuropathologies [17, 34, 37]. These findings suggest that conformation of α-synuclein might have some role in determining disease type.

Cell-to-cell transmission of pathogenic α-synuclein is thought to be associated with disease progression in synucleinopathies [38]. Although a comprehensive mechanism has not been elucidated, recent studies demonstrated the importance of α-synuclein recognizing receptors and the mass action of α-synuclein aggregates driven by genetic defects, protein homeostasis impairment, and cellular stress such as oxidative stress [39, 40]. Once pathological α-synuclein is transmitted to neighboring neurons, they can trigger the misfolding of endogenous neuronal α-synuclein which may consequently lead to neurotoxic cellular damage via mitochondrial dysregulation, the disruption of vesicle trafficking, and impairment of protein quality control [41, 42].

In addition to α-synuclein autonomous neurotoxicity during pathogenesis, non-autonomous biological properties of structurally distinct α-synuclein aggregates, such as α-synuclein polymorphs-mediated neuroinflammation may contribute significantly to disease progression. However, these mechanisms remain poorly understood. Growing evidence indicates the presence of structurally heterogeneous α-synuclein aggregates in the brains of patients with synucleinopathies [33–36]. Although some studies have isolated theses aggregates and investigated their biological properties, reproducing their structural characteristics has been challenging due to technical limitations [43]. As an alternative, numerous studies have employed recombinant proteins or neuronal cell models to generate structurally distinct α-synuclein aggregates, aiming to infer their potential roles in the disease pathogenesis. In the present study, we utilized various types of homogenous α-synuclein aggregates to compare their roles in microglial neuroinflammation.

Neuroinflammation is another key pathological feature of synucleinopathies and deposition of α-synuclein aggregates play important role in this process. One proposed mechanism involves the interaction of α-synuclein with various pattern recognition receptors (PRRs), which activate neuroinflammatory pathways. We previously demonstrated that neuron-released, β-sheet enriched oligomeric forms of α-synuclein directly interact with the PPR TLR2, thereby inducing neurotoxic microglial inflammation [12, 44]. TLR4 is also known to recognize pathogenic α-synuclein aggregates, and genetic depletion of TLR4 ameliorates neuroinflammation and neurodegeneration in PD mouse models [14, 45]. In addition to TLR2 and 4, several other receptors, including MAC-1, β1-integrin, NADPH oxidase, CD36, heparan sulfate, N-methyl-D-aspartate receptor (NMDAR), Neurexin-α, metabotropic glutamate receptor 5 (mGluR5), Fc fragment of IgG receptor IIb (FCGR2B), and P2X purinoceptor 7 (P2X7) have been identified as potential interactors of α-synuclein aggregates [46–53]. However, most studies have employed a limited range of α-synuclein aggregate forms and have not adequately considered their receptor recognition profiles. Additionally, the field lacks standardization in the classification and characterization of theses α-synuclein aggregates. In the present study, we aimed to provide detailed structural characterization of various α-synuclein aggregates and to evaluate their abilities to activate microglia via TLRs. We found that α-synuclein KSO, sPFF, and Fib induced expressions of proinflammatory cytokine and chemokine genes in microglia, whereas EO, DO, and PFF did not elicit such responses. Transcriptome analysis further revealed that all α-synuclein forms were capable of triggering microglial immune responses, albeit to varying extents. These modest but detectable inductions may be particularly relevant in the context of the chronic and progressive nature of neuroinflammation observed in synucleinopathies. Radar plot analysis of KEGG pathways demonstrated that different α-synuclein aggregates elicited distinct transcriptional responses in microglia. Furthermore, we confirmed that α-synuclein KSO exhibits agonistic activity toward both TLR2 and TLR4, while only sonicated PFF demonstrated TLR4 agonist activity.

Treatment with different α-synuclein polymorphs elicited generally similar inflammatory responses in iMG. However, transcriptomic DEGs enrichment analysis revealed distinct signatures of cellular signaling pathways. Exposure to α-synuclein polymorphs commonly induced the chemokine signaling pathway, while treatment with KSO, PFF, and Fib resulted in greater enrichment of the TNF signaling pathway. Notably, the C-type lectin receptor signaling pathway was specifically enriched in iMG exposed to α-synuclein KSO and Fib, but not by other polymorphs. Further analysis of DEGs related to cellular processes showed that α-synuclein Fib elicited a distinct transcriptional response in iMG compared to other polymorphs, particularly in pathways associated with cytokine response, inflammatory response, and defense mechanisms. Although we were unable to pinpoint specific cellular mechanisms underlying the differential responses to each polymorph, we demonstrated that certain α-synuclein conformations can activate Toll-like receptors (TLRs). These findings are significant, as physiological forms of α-synuclein aggregates are known to interact with TLRs [12, 54]. However, α-synuclein may also interact with additional immune cell receptors. Therefore, further detailed studies are necessary to develop a comprehensive understanding of how α-synuclein polymorphs contribute to microglia-mediated neuroinflammation.

Exposure to α-synuclein polymorphs induced the expression of inflammatory genes in iMG following short-term treatment (Fig. 1). To validate these findings, we examined mouse primary microglia and observed that α-synuclein polymorphs increased inflammatory gene expression only after prolonged exposure (24 h) (Supplementary Fig. 4). In contrast, previous studies have shown that primary mouse microglia mount robust inflammatory responses even after short-term exposure to neuron-released α-synuclein or other stimuli [12, 55]. We speculate that this discrepancy may be due to differences in both the amount and nature of the stimuli. For instance, neuron-released α-synuclein likely contains a higher proportion of oligomeric species with TLR-agonist-like activity, and there may be differential expression of receptors that recognize α-synuclein polymorphs.

While our investigation provides valuable insights into the role of α-synuclein polymorphs in microglial neuroinflammation, it is important to acknowledge a key limitation: the use of recombinant α-synuclein. Despite offering advantages in reproducibility and standardization, recombinant forms may not fully capture the complexity of physiological aggregates found in the brains of synucleinopathy patients. Future studies utilizing patient-derived α-synuclein aggregates will be essential to further elucidate the non-cell-autonomous roles that these polymorphs may play in disease progression.

## Supplementary Information

Below is the link to the electronic supplementary material.

Supplementary Material 1

## Data Availability

The RNAseq data generated in this study have been deposited in the Gene Expression Omnibus GEO of NCBI under the accession number GSE305401. The data that support the findings of this study are available from the corresponding author upon reasonable request.
