# Supplementary material for: Differential microglial responses to structurally distinct alpha-synuclein polymorphs
Source: Mol Brain. 2025 Dec 5;19:3. doi: 10.1186/s13041-025-01256-0 (PMC12797977; doi:10.1186/s13041-025-01256-0)
Supplement: Supplementary file 1 — Supplementary Material 1 [file 13041_2025_1256_MOESM1_ESM.pdf]

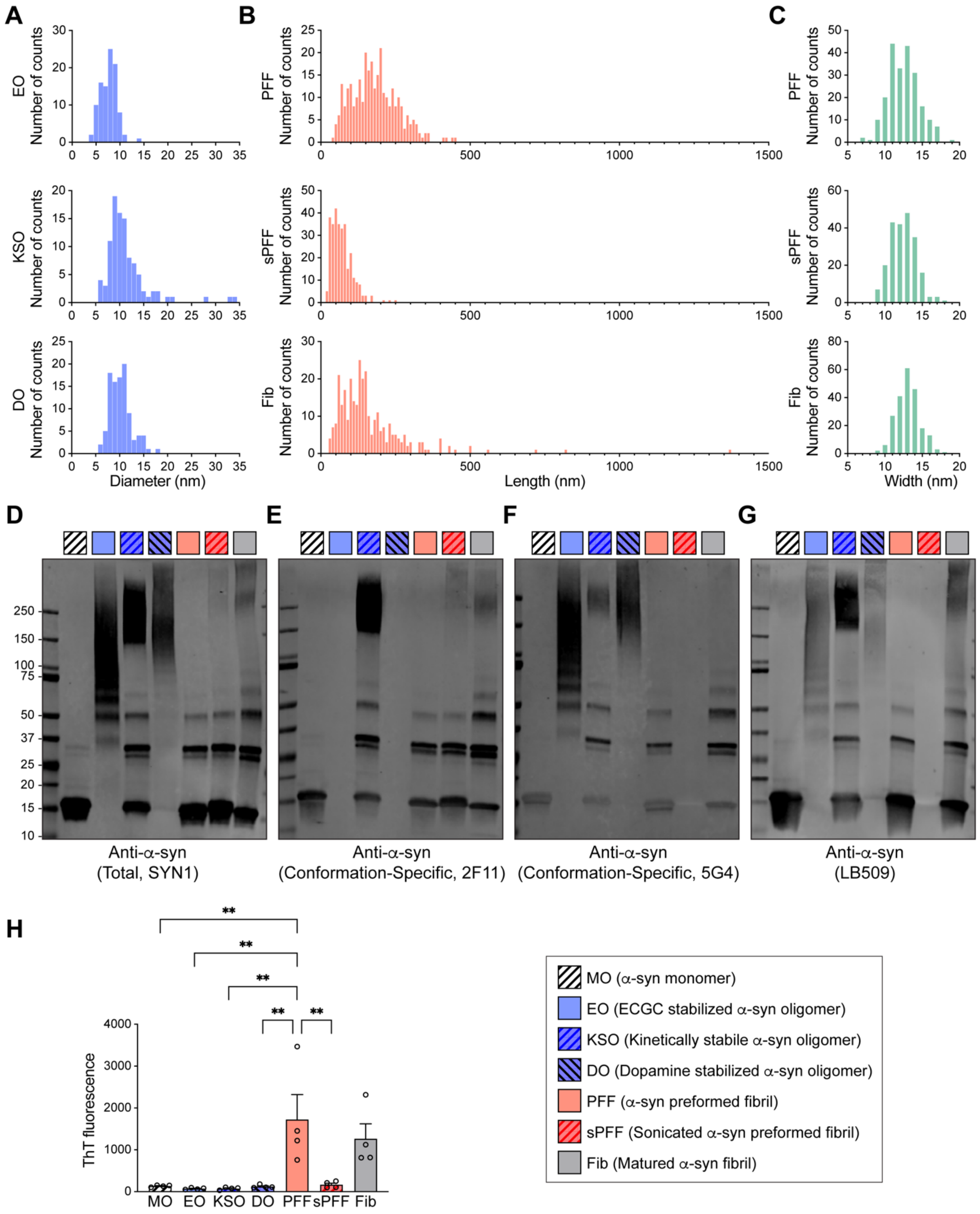

A
B
C
EO
KSO
DO
PFF
sPFF
Fib
Number of counts
Diameter (nm)
Length (nm)
Width (nm)
D
E
F
G
Anti-α-syn
(Total, SYN1)
Anti-α-syn
(Conformation-Specific, 2F11)
Anti-α-syn
(Conformation-Specific, 5G4)
Anti-α-syn
(LB509)
H
ThT fluorescence
MO EO KSO DO PFF sPFF Fib
MO (α-syn monomer)
EO (ECGC stabilized α-syn oligomer)
KSO (Kinetically stabile α-syn oligomer)
DO (Dopamine stabilized α-syn oligomer)
PFF (α-syn preformed fibril)
sPFF (Sonicated α-syn preformed fibril)
Fib (Matured α-syn fibril)

Supplementary Fig. 1 Structural analysis of α-syn aggregates. (**A-C**) Assessments of the sizes of α-synuclein aggregates. The diameter of α-synuclein oligomers (**A**), the lengths of α-synuclein fibrillar species (**B**), and the widths of α-synuclein fibrillar species (**C**) were displayed with distribution histograms (*n* = 150, 300, 218, respectively). (**D-G**) Immunoblot analysis of α-synuclein aggregates. The sample blots were probed with total (SYN1) or α-synuclein conformational specific antibodies (2F11, 5G4, and LB509). (**H**) Amyloid fibril structure of α-syn aggregates was determined by Thioflavin T (ThT) assay (*n* = 4 per group). Error bars represent SEM. One-way ANOVA and Tukey's multiple comparison test. $^{**}p < 0.01$.

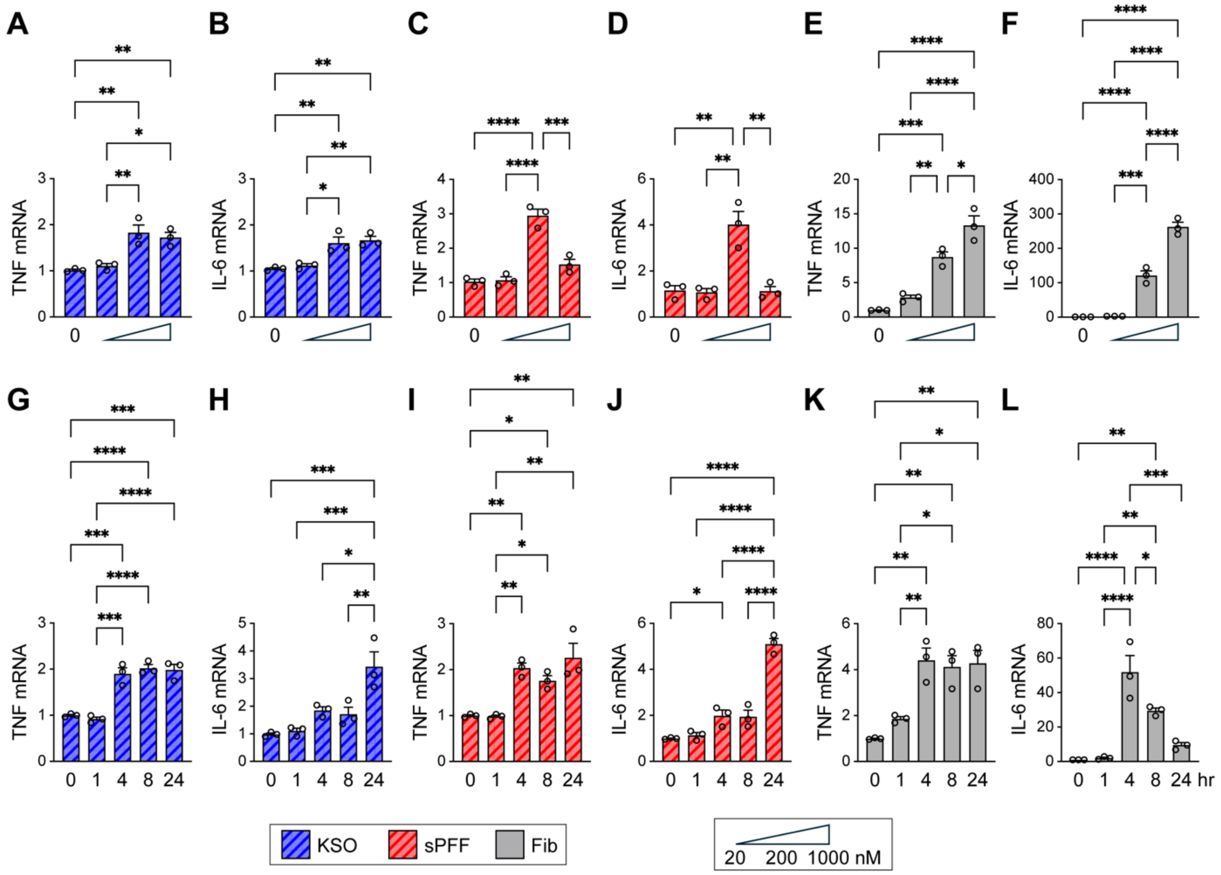

Supplementary Fig. 2 Dose- and time-dependent induction of inflammatory cytokine expression in iMicroglia by α-synuclein polymorphs. (**A-F**) iMG were treated with the indicated concentrations of α-synuclein KSO (**A, B**), sPFF (**C, D**), and Fib (**E, F**). Expression levels of TNF and IL-6 were assessed by quantitative PCR. (**G-L**) iMG were treated with of α-synuclein KSO (**G, H**), sPFF (**I, J**), and Fib (**K, L**) for indicated hours. Expression levels of TNF and IL-6 were assessed by quantitative PCR. Error bars represent SEM. One-way ANOVA and Tukey's multiple comparison test (n = 3 per group). *$p < 0.05$, **$p < 0.01$, ***$p < 0.001$, ****$p < 0.0001$.

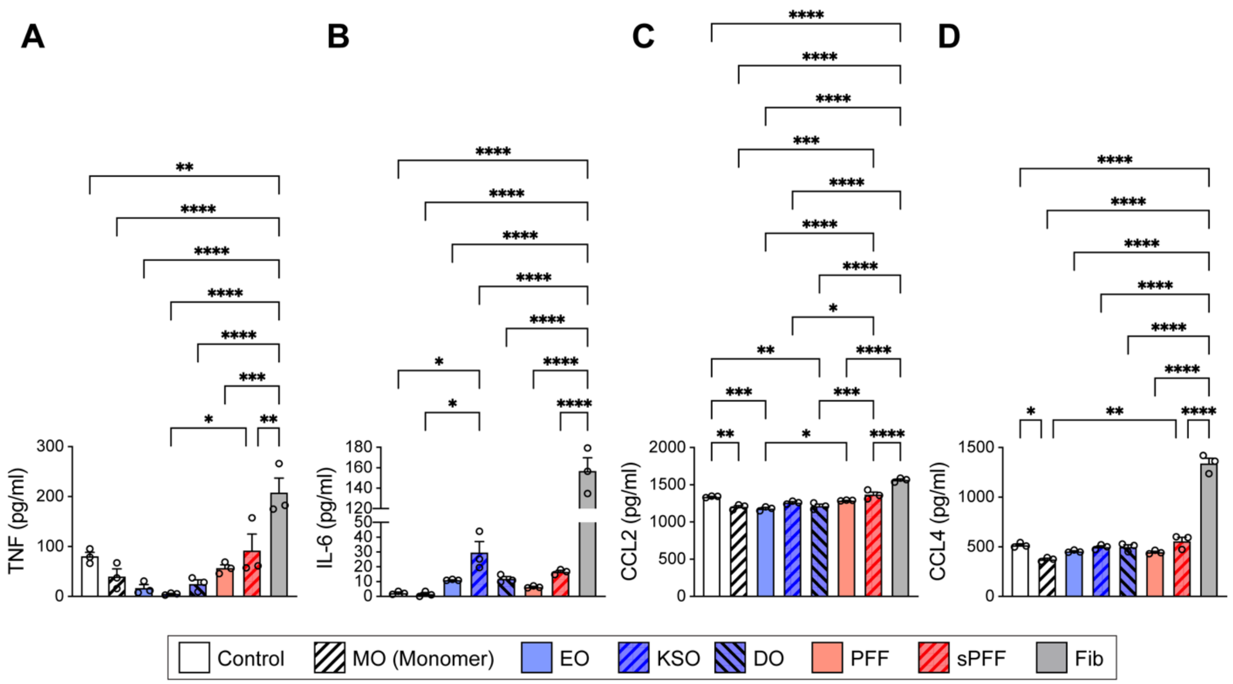

Supplementary Fig. 3 iMicroglial secretion of inflammatory cytokines and chemokines in response to α-synuclein polymorphs. iMG were treated with control, α-synuclein MO, EO, KSO, DO, PFF, sPFF, or Fib for 24 hours. (**A-D**) Secretion levels of TNF (**A**), IL-6 (**B**), CCL2 (**C**), and CCL4 (**D**) were measured by ELISA assay (n = 3 per group). Error bars represent SEM. One-way ANOVA and Tukey's multiple comparison test. *$p < 0.05$, **$p < 0.01$, ***$p < 0.001$, ****$p < 0.0001$

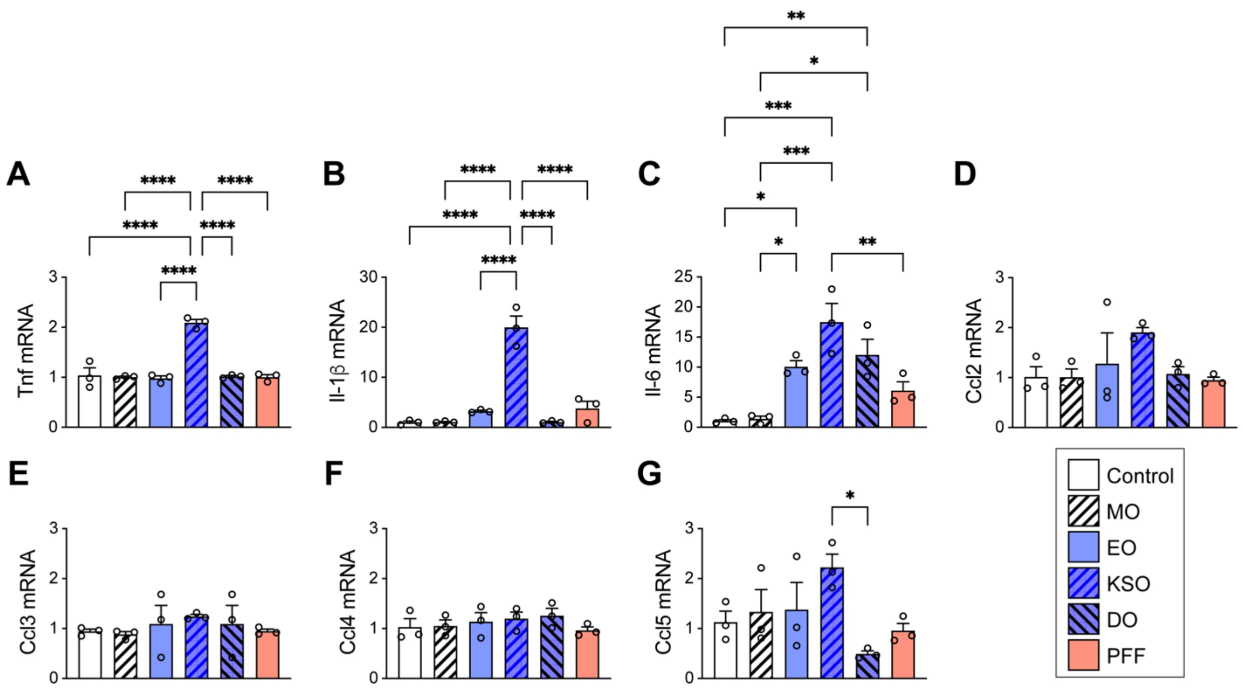

Supplementary Fig. 4 Pro-inflammatory gene expressions in primary mouse microglia. Primary mouse microglia were treated with control, α-synuclein MO, EO, KSO, DO, or PFF for 24 hours. (**A-G**) The expressions of pro-inflammatory cytokines/chemokines, Tnf (**A**), Il-1β (**B**), Il-6 (**C**), Ccl2 (**D**), Ccl3 (**E**), Ccl4 (**F**), and Ccl5 (**G**), were determined by quantitative PCR ($n = 4$ per group). Error bars represent SEM. One-way ANOVA and Tukey's multiple comparison test. *$p < 0.05$, **$p < 0.01$, ***$p < 0.001$, ****$p < 0.0001$

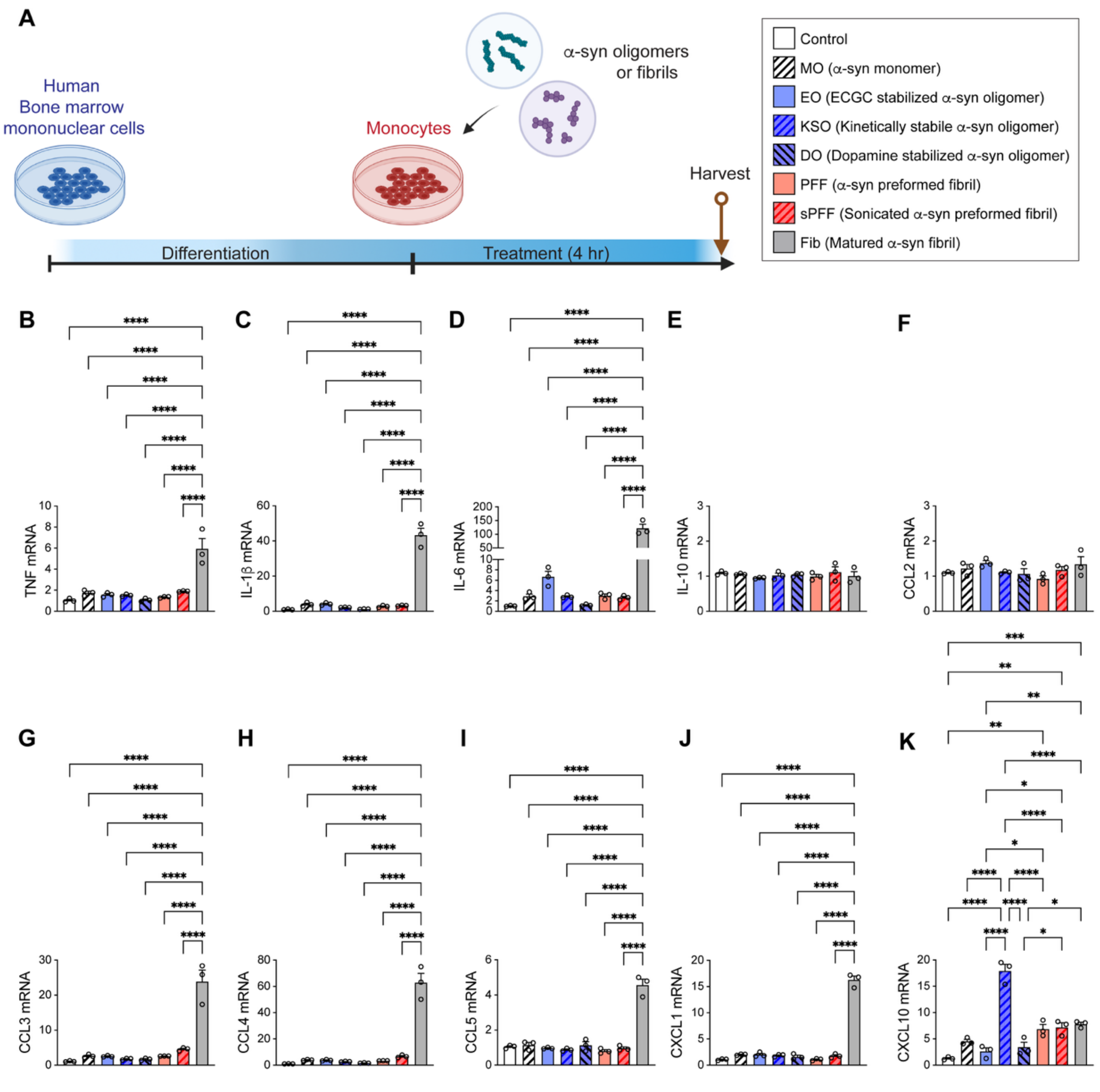

Supplementary Fig. 5 Inflammatory gene expression in monocytes induced by α-synuclein polymorphs. (**A**) Human bone marrow mononuclear cell-derived monocytes were treated with control, α-synuclein MO, EO, KSO, DO, PFF, sPFF, or Fib for 4 hours. (**B-K**) Expression levels of TNF (**B**), IL-1β (**C**), IL-6 (**D**), IL-10 (**E**), CCL2 (**F**), CCL3 (**G**), CCL4 (**H**), CCL5 (**I**), CXCL1 (**J**), and CXCL10 (**K**) were determined by quantitative PCR. Error bars represent SEM. One-way ANOVA and Tukey's multiple comparison test (n = 3 per group). *$p < 0.05$, **$p < 0.01$, ***$p < 0.001$, ****$p < 0.0001$.

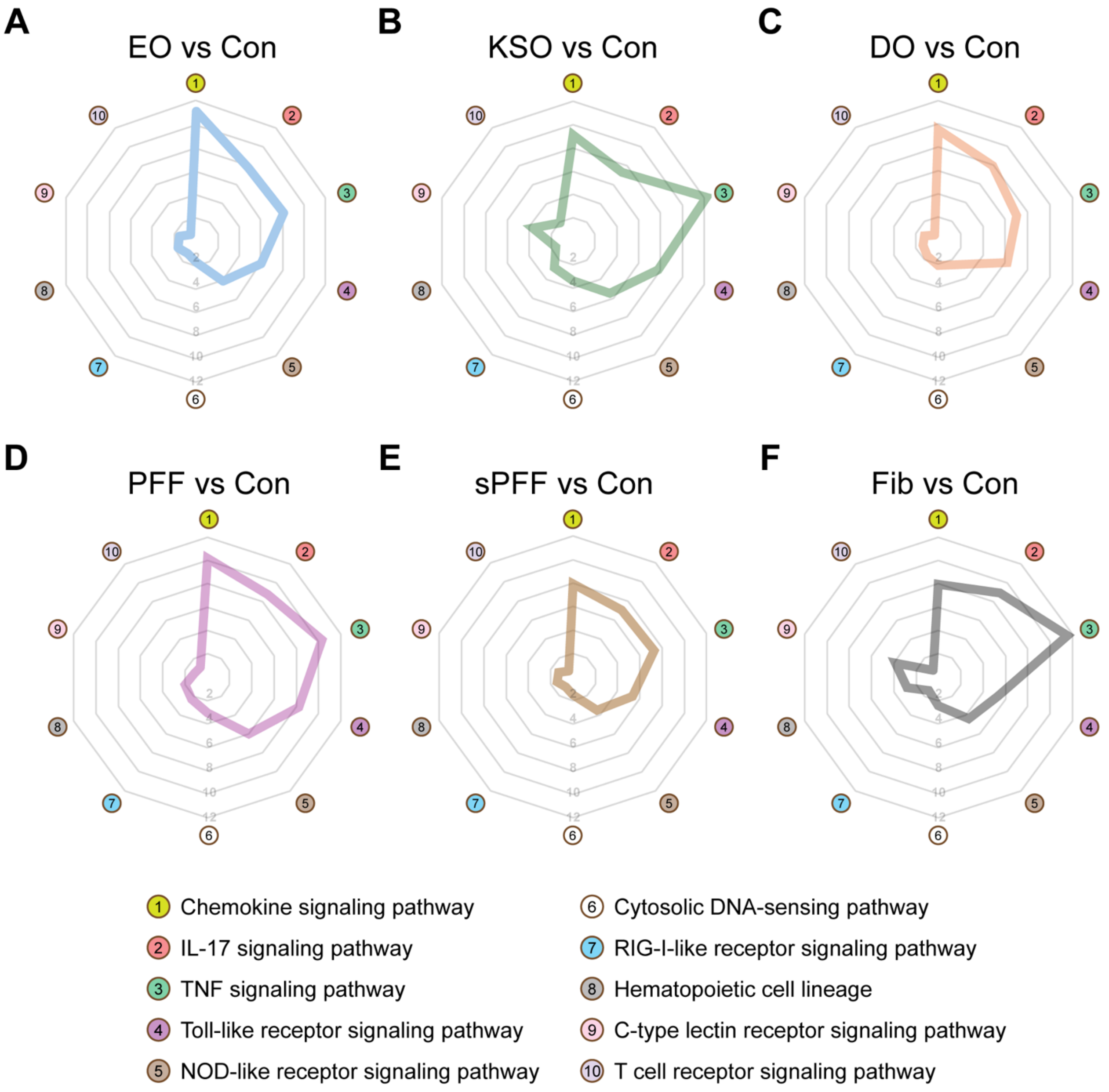

Supplementary Fig. 6 Radar plot mapping of iMicroglial transcriptome exposed to α-synuclein polymorphs. Enriched KEGG immune pathways in iMG exposed to α-synuclein aggregates were analyzed by radar plot analysis. (**A-F**) Chemokine signaling pathway, IL-17 signaling pathway, TNF signaling pathway, toll-like receptor signaling pathway, NOD-like receptor signaling pathway, cytosolic DNA-sensing pathway, RIG-I-like receptor signaling pathway, Hematopoietic cell lineage, C-type lectin receptor signaling pathway, and T cell receptor signaling pathway of comparisons control versus EO (**A**), KSO (**B**), DO (**C**), PFF (**D**), sPFF (**E**), and Fib (**F**).

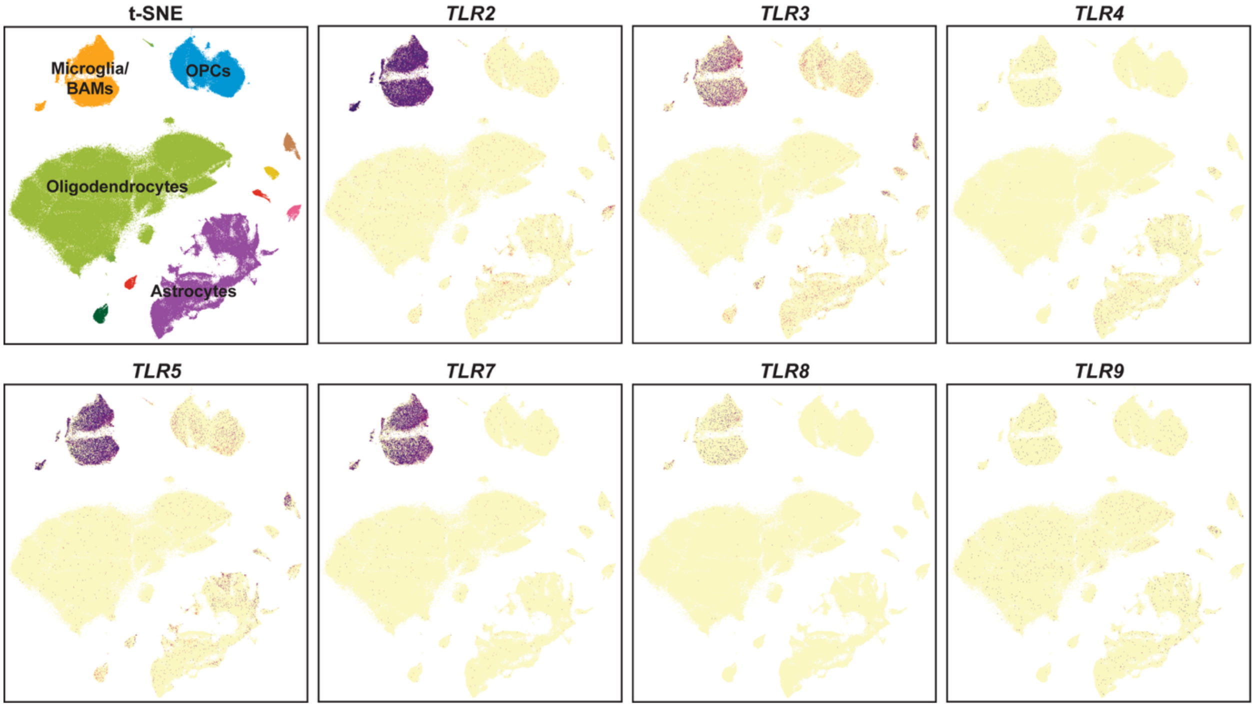

Supplementary Fig. 7 TLR expressions in non-neuronal cells residing in the human brain. Images were gathered from the Allen Brain Cell Atlas, specifically human single-nucleus RNA sequencing data from Siletti et al., 2023 [1]. Cells shown are derived from around 100 dissections spanning the whole human brain from three donors. Images show broad cell-type labels ("t-SNE") or relative expression of toll-like receptor genes across non-neuronal cell types. Gene expression is depicted as $\log_2$ (counts per million + 1), with relative color scaling for each gene; darker coloring depicts higher gene expression. Image credit: Allen Institute for Brain Science (https://knowledge.brain-map.org/abcatlas).

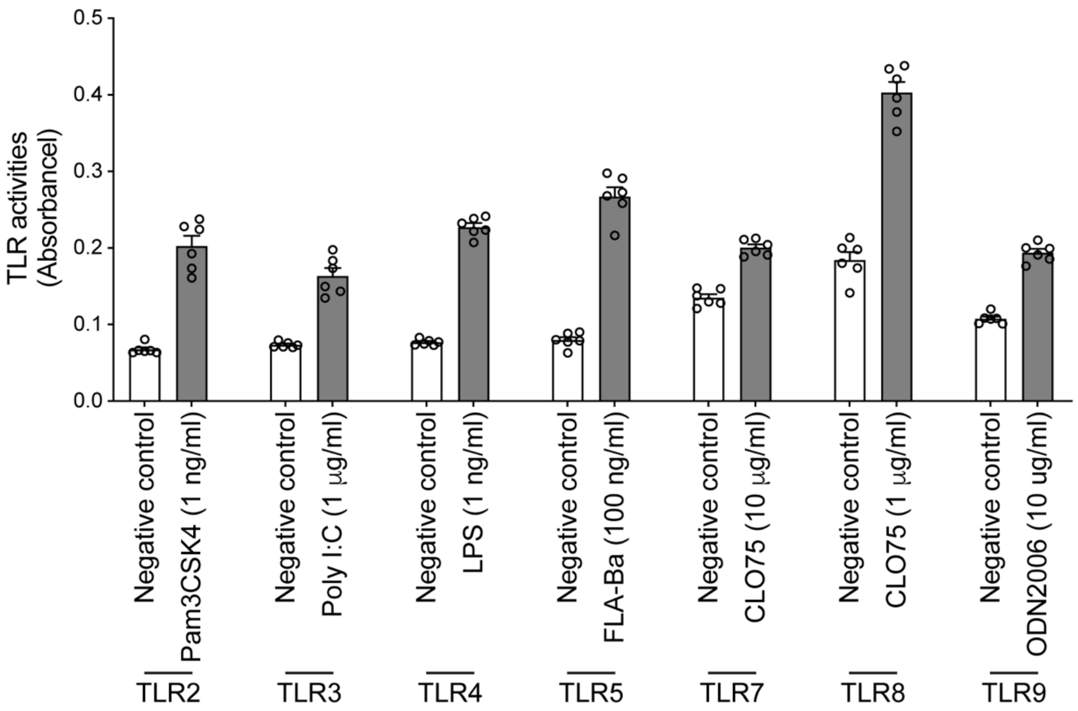

Supplementary Fig. 8 Validation of HEK-Blue hTLR reporter. HEK-Blue hTLR reporter cells were exposed to vehicle or indicated concentration of agonist for 24 hours. TLR activities were determined by TLR reporter assay (*n* = 6 per group).

Supplementary Table 1 List of antibodies for current study. The table shows comprehensive information on antibodies used. From left to right: Target gene, specificity, host species, dilutions, source, catalog number, and notes as applicable.

**Antibody**	**Host species**	**Dilution**	**Source**	**Cat NO.**	**Note**
Anti-α-syn (SYN1)	Mouse	1:1K	BD Biosciences	610797	Primary
α-syn (2F11)	Mouse	1:1K	StressMarq	SMC-617	Primary
α-syn (5G4)	Mouse	1:1K	Millipore Sigma	MABN389	Primary
α-syn (LB509)	Mouse	1:1K	Abcam	MS- ab27766	Primary
IRDye® 800CW Goat anti-Rabbit IgG (H + L)	Goat	1:20K	LI-COR Biosciences	925-32211	Detection
IRDye® 800CW Goat anti-Mouse IgG (H + L)	Goat	1:20K	LI-COR Biosciences	925-32210	Detection
IRDye® 680RD Goat anti-Rabbit IgG (H + L)	Goat	1:20K	LI-COR Biosciences	925-68071	Detection
IRDye® 680RD Goat anti-Mouse IgG (H + L)	Goat	1:20K	LI-COR Biosciences	925-68070	Detection

Supplementary Table 2 List of reagents for iPSC culture. The table shows information on iPSC culture. From left to right: Target gene, Source, and Catalog number.

Reagents	Source	Cat NO.
Essential 8™ Medium	Thermo Fisher Scientific	A1517001
Supplement	Thermo Fisher Scientific	A15171–01
ROCK inhibitor	StemCell Technologies	72307
Ultra-low attachment 96-well plates	S-bio	MS-9096VZ
BMP-4	Thermo Fisher Scientific	120-05ET
SCF	Thermo Fisher Scientific	300-07
VEGF-121	Thermo Fisher Scientific	100-20A
VWR® Wide Bore Pipette Tips	Avantor, Inc	76635-650
X-VIVO 15	Lonza	04-418Q
M-CSF	Thermo Fisher Scientific	300-25
IL-3	Thermo Fisher Scientific	200-03
Fibronectin	Sigma	F0895
RPMI 1640 Medium	Thermo Fisher Scientific	12633012
GlutaMax	Thermo Fisher Scientific	35050061
IL-34	Thermo Fisher Scientific	200-34
GM-CSF	Thermo Fisher Scientific	300-03

Supplementary Table 3 List of primers for quantitative PCR. The table shows information on primers. From left to right: Target gene, Source, and Catalog number.

Target gene	Source	Cat NO.
Human TNFα	Thermo Fisher Scientific	Hs00174128
Human IL-1β	Thermo Fisher Scientific	Hs01555410
Human IL-6	Thermo Fisher Scientific	Hs00174131
Human IL-10	Thermo Fisher Scientific	Hs00961622
Human CCL2	Thermo Fisher Scientific	Hs00234140
Human CCL3	Thermo Fisher Scientific	Hs00234142
Human CCL4	Thermo Fisher Scientific	Hs99999148
Human CCL5	Thermo Fisher Scientific	Hs00982282
Human CXCL1	Thermo Fisher Scientific	Hs00236937
Human β-actin	Thermo Fisher Scientific	Hs03023880
Mouse Tnfα	Thermo Fisher Scientific	Mm00443258
Mouse Il-1β	Thermo Fisher Scientific	Mm00434228
Mouse Il-6	Thermo Fisher Scientific	Mm00446190
Mouse Ccl2	Thermo Fisher Scientific	Mm00441242
Mouse Ccl3	Thermo Fisher Scientific	Mm99999057
Mouse Ccl4	Thermo Fisher Scientific	Mm00443111
Mouse Ccl5	Thermo Fisher Scientific	Mm01302427
Mouse β-actin	Thermo Fisher Scientific	Mm00607939

Supplementary Table 4 List of agonists for TLRs. The table shows the information on primers. From left column to right: Target gene, Source, and Catalog number.

Agonist	TLRs	Concentration	Source	Cat NO.
Pam3CSK4	2	1 ng/ml	InvivoGen	tlrl-pms
Poly I:C	3	1 µg/ml	InvivoGen	tlrl-pic
LPS	4	1 ng/ml	InvivoGen	tlrl-rslps
FLA-BS	5	100 ng/ml	InvivoGen	tlrl-bsfla
CLO75	7/8	10 µg/ml (TLR7)<br>1 µg/ml (TLR8)	InvivoGen	tlrl-c75
ODN2006	9	10 µg/ml	InvivoGen	tlrl-2006

Supplementary reference

1. Siletti K, Hodge R, Mossi Albiach A, Lee KW, Ding SL, Hu L, Lonnerberg P, Bakken T, Casper T, Clark M, et al: **Transcriptomic diversity of cell types across the adult human brain.** *Science* 2023, **382:**eadd7046.
